# “A Whole New World of Possibilities”: Koji Uses and Ambiguities on the Global Marketplace

**DOI:** 10.1080/15528014.2024.2447663

**Published:** 2025-01-09

**Authors:** Maya Hey, Eleni Michael

**Affiliations:** a Department of Sociology, University of Helsinki, Finland

**Keywords:** Koji, enzymes, microbes, material-discursive formations, gourmandization

## Abstract

This article analyzes the ambiguity of the term “koji” – as it simultaneously describes a mold spore, a set of enzymes, and a fermented ingredient – across products that make, use, and promote koji in an increasingly globalized marketplace. We focus on three case studies – a koji-based chocolate, a lees-based condiment with koji in its name, and a hot sauce that is doubly fermented with koji – using discourse analysis supplemented by interviews. As practitioners and scholars who work with koji, we are keen to disentangle how koji is used literally and conceptually across new contexts because slippages between koji-the-spore, koji-the-enzymes, and koji-the-ingredients enable an ambiguity that allows the gourmandization of koji to thrive in capitalism. With attention to koji’s material-discursive properties, this paper analyzes the ambiguity of koji across different places and languages. Our findings point to themes of selective storytelling, social distinction, and human-microbe labor relations, which feed into each other.

## Introduction: koji and its fermentative possibilities

Koji has historically been used to ferment key condiments in the Japanese cuisine, including miso, sake, and shoyu. In recent times, koji has been spreading globally, partly because of its enzymatic potential. For instance, a common refrain describes koji as a “powerhouse of enzymes” (Shih and Umansky 2020, 5) given the high concentration of amylases (breaking down starches into fermentable sugars) and proteases (breaking down proteins into amino acids). These enzymes have expanded the use-cases beyond Japanese cuisine, enabling innovations that enhance umami-based flavors and/or create tastes using less resource-intensive means (e.g., koji-aged charcuterie that mimics the flavor development of dry-aging meats, a chocolate alternative made from cereals and legumes using koji). We also see the increasing appearance of fermentation-led Research and Development (R&D) departments in fine-dining restaurants with subsequent dishes and products made with koji (e.g., Noma in Copenhagen, Momofuku in New York, and Mugaritz in the Basque Country). It is an opportunistic time for koji, supported by the wider social awareness of fermentative possibilities. But it is also a time for confusion and misinformation, especially in the colloquial broken telephone that makes up the knowledge transfers from one fermentation practice to another.

This paper scrutinizes the material-discursive practices of koji usage to parse out when, how, and in what configurations koji comes to matter. By analyzing three case studies, the paper examines the ambiguity with the term “koji” – as it simultaneously describes a mold spore, a set of enzymes, and a fermented ingredient – with a particular focus on how this ambiguity, for better or for worse, thrives in a capitalist context.

We contend that this ambiguity is worth scrutinizing for three inter-related reasons. First, the enthusiasm for koji seems to leverage sustainable food production and the so-called “future of food.” Yet, while koji in restaurants and novel products can elevate, transform, and innovate food for someone’s future, the ambiguities of what exactly koji *is* or *does* ushers in assumptions and narratives that allow only certain people to make use of and lay claim to the ferment. By providing tangible examples of international(−ized) uses of koji, we critically examine the (at times discordant) material, discursive, and relational effects that come with the sprawl and valorization of ferments. As such this paper is not about nomenclature; it is about what allows certain koji practices – materially, discursively – to come forth, persist, and lay unquestioned, especially against the backdrop of fermentation hype. Lastly, the practitioners we study are also *choosing* to ferment with koji, having at least some wherewithal to experiment and fail. This makes their actions and how they share their knowledge about koji important for the social, cultural, ideological shifts in the current fermentation revival and greater foodscape.

In what follows, we first locate koji matters in existing discourses and practices, followed by three case studies that use koji: a chocolate, a mayonnaise, and a hot sauce. We follow with a thematic analysis, explaining how strategic storytelling, the gourmandization of koji, and human-microbe labor relations shape koji practices. We conclude with opportunities for further research and name the stakes at hand.

## Situating Koji: background to the term and foodstuff

Koji is multiplicitous, representing a range of ferments. For one, koji-making commonly relies on the fungus *Aspergillus oryzae* but can also refer to subspecies including *Aspergillus kawachii, Aspergillus luchuensis*, and *Aspergillus sojae*, depending on intended ferment (e.g., sake versus the distilled beverage shochu) or regional difference (e.g., ferments in Chinese, Korean, and Thai contexts).

In Japanese contexts, *A. oryzae* is often synonymous with koji because it was declared Japan’s National Mold (Ichishima 2020); however, the exclusive claims of koji as an indigenous mold to Japan remain questionable given the diverse fermentation practices that span East Asia (Hong, Kim, and Samson 2015; Katz 2012, 2021; Shih and Umansky 2020). Instead, koji’s discovery and subsequent domestication may be characterized as “a happy accident” (Rath 2023, 35) that has since been capitalized upon, both economically and culturally (Lee 2021). For most applications, making koji entails inoculating rice and/ or legumes with the fungus *A. oryzae*, and the resulting koji can be further used to make seasonings like sake, miso, and shoyu. That said, koji for sake-making differs from koji in shoyu-making, which can differ still in other applications (e.g., shochu brewing) – these differences inhere not just in fungal species but also in substrates (e.g., rice, wheat, and beans). So even though koji is a Japanese term linguistically, *materially* koji can refer to many different foodstuffs even within the confines of Japanese cuisine.

Historical studies offer insights into how Japan domesticated *Aspergillus* and subsequently nationalized it as koji (Lee 2019; Rath 2023). The use of the *Aspergillus* family ranges from staple fermented foods of East Asian cultures such as miso and amazake (Ogura 2017; Rath and Watanabe 2023) to being deployed at an industrial scale to produce flavor enhancers like monosodium glutamate today (Laurent 2022; Tracy 2019). This trajectory marks the shift from its early uses for household consumption to its later adoption as an instrument of “metabolic engineering” in food laboratories (Lee 2021, 271). Across centuries, the throughline for the fungus (in Japan at least) is its utility; it has been and will continue to be an enabler of human cultures, even if imposing increasingly modern methods onto it.

In recent decades, resources in English have made koji knowledge accessible to increasingly wider audiences, with publications for professional settings (Redzepi and Zilber 2018; Shih and Umansky 2020; Shurtleff and Aoyagi 1976) as well as household settings (Groen 2021; Katz 2003; Nakaji 2020; Shockey and Shockey 2019). In parallel, practitioners from a global culinary community have apprenticed in a variety of koji-making settings such as sake breweries and miso fermentaries in Japan, embodying the know-how and returning to their respective countries to apply their learnings. Or, independent practitioners will lead courses and workshops for individuals (e.g., Ken Fornataro) in a way that makes tracing the lineage of koji knowledge possible (e.g., Nakaji taught Haruko Uchishiba of Koji Fermentaria, who taught chef Sam Black, who now offers courses via Patreon). Place-based fermentation hubs (e.g., Hakko Department in Tokyo) and online hubs (e.g., KojiCon) also double as platforms for workshops, tours, and lectures. This range of koji sources have engaged people from diverse backgrounds to experiment with koji, spreading its know-how across different countries and circumstances.

As practitioners and scholars who take up koji as a site of inquiry, we are keen to clarify how, exactly, koji figures in contemporary foodways in material-discursive ways.

## Taking a material-discursive approach to studying Koji

We employ a mixed theoretical framework that examines human and nonhuman entanglements. On the one hand, we understand knowledge production in food studies to be always partial (Haraway 1991), always relational (Mol 2021), and always a part of an ecology of practices (Stengers 2010). Relying on a feminist foundation helps us see the people in-and-around koji to be situated and tethered to social, material, and place-based attachments that make each case study specific and grounded in the real. On the other hand, we use material-discursive formations to guide our inquiry and direct our attention to words *and* the practices that they describe, precisely because “language has been granted too much power” (Barad 2003, 801). We use this particular flavor for studying discursive formations to shore up “a robust account of the materialization of *all* bodies – ‘human’ and ‘nonhuman’ – and the material-discursive practices by which their differential constitutions are marked” (810, original emphasis). Material-discursive, in this sense, pertains to the material, microbial, environmental, and biophysical realities and their inherent co-constitution with ideologies, ethics, and cultural practices. Here, we see language as one key practice where how one engages with the world and describes it inherently draws boundaries around what matters and what gets excluded (Giraud 2019). We also follow the narratives of koji, in that it matters what stories we tell and which connective ties we use in analysis (Haraway 2016, 12). We see this mixed approach as necessary for understanding the uses of koji in material/literal and discursive/conceptual ways.

This paper analyzes three case studies selected based on four criteria: koji (1) is used in a novel setting, (2) it remains common or recognizable to the everyday eater, allowing it to help reimagine or reposition the foodstuff, (3) it is framed in a disruptive, future-proof, or forward-thinking manner, and (4) it is designed for a globalized foodscape. Despite the global reach, we focused on products that were marketed in English (alongside other languages) for consistent analysis; any other language was one that was professionally or natively familiar to us. Of the five potential cases, we selected three based on their differences in company size, production scale, the leaders behind the innovation, and their distinct locations across continents. We decided to exclude restaurants, their dishes, and their recipes due to their ephemerality and instead directed our attention toward products and their messaging, effectively “reading” products as “texts” unto themselves. We arrived at three case studies – a chocolate, a mayonnaise, and a hot sauce – which span three countries for production. The remainder of this paper is structured around these three case studies.

Methodologically, this paper uses discourse analysis and examines both the written (e.g., brand messaging) and the spoken (e.g., interviews). Discourse analysis allows for “the close study of talk (or text) in context [to] make sense of social members’ ongoing interactional practices” (Tracy 2001, 734). In each case study, we attended to the historically contingent, social construction of meanings in interviews of 60 min (totaling five) and publicly available texts (e.g., websites, articles, and food labels). Interviews were transcribed and analyzed with particular attention to word choice “as a form of social practice” whereby words simultaneously enact “‘ideational,’ ‘interpersonal,’ and ‘textual’ functions of language” (Fairclough 1995, 131). When appropriate, interview questions inquired about the material practices and hands-on processes for making the koji-based product. All data were coded using thematic analysis, with attention to what “represents some level of *patterned* response or meaning within the data set” (Braun and Clarke 2006, 82, original emphasis). Of the five themes coded, we present three patterns in this paper (the two omitted were considered out of scope for food studies). We chose to focus on the patterns of slippage and the ambiguity in koji’s newer use-cases to better understand its social, cultural, and microbial implications.

## Case study 1. Koji Fermented Cacao by Subko

Subko Specialty Coffee Roasters and Craft Bakehouse in India has been developing a novel koji-fermented chocolate. For comparison, conventional chocolate production relies on acid-producing bacteria and yeast to convert the cacao bean into what we know as chocolate, whose metabolic processes release esters and other aromatic compounds that make up the characteristics that become notable in bean-to-bar chocolates (Schwan and Wheals 2004). So, barring a few contemporary examples (e.g., Niida Honke in Japan, Chocobien in Hong Kong), fermenting cacao pods with koji is – as of this writing – rare.

Founded in 2020 in Mumbai, Subko focuses on craft coffee and chocolate sourced from key regions in India. While it first began with only one outlet in Mumbai, it has now grown to almost 200 employees with local recognition for their specialty coffee (Chatterjee 2022; Chowdhury 2020) and their bean-to-bar chocolate program (Mehta 2024). Their koji-fermented product line was developed by Prasanna Gudi, Director of Coffee at Subko, who leads product innovation.

Gudi notes that koji fermentation started in the broader international coffee scene around 2019, led by coffee specialist Christopher Feran. Intrigued, Gudi recalls testing koji on his own coffee: “We did like two, three experiments on this until we got *good results*. Because, you know, that was new for us” (pers. comm., October 31, 2023, emphasis added). For Gudi, “good results” were characterized by flavor outcomes which were novel and complex. His experiment with coffee led him to apply this technique to Subko’s other commodity: “why not koji on cacao?” He credits koji as being the driving force behind innovation, stating that “the thing with koji is that it sparkles you [sic] on different ideas” (pers. comm., October 31, 2023).

**Figure 1:**
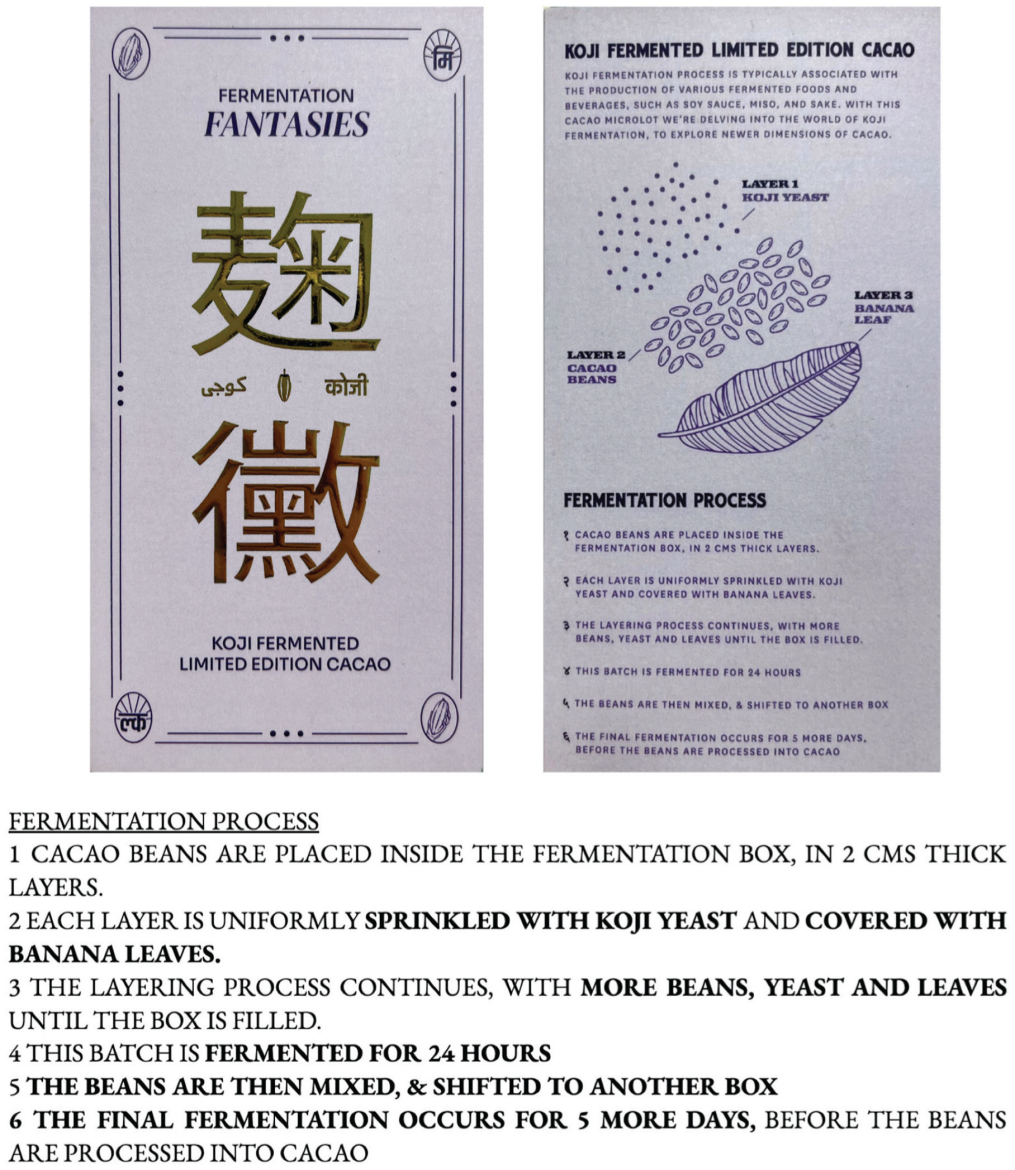
Packaging of koji-fermented limited edition cacao.

Subko prominently features koji on their package (Figure 1) with explicit mention of its English name as well as the kanji character (麹). Gudi clarifies that the chocolate label intentionally adds Japanese elements to a product made in India, “as a way of connecting India and Japan” (pers. comm., October 31, 2023).^1^ By incorporating koji into contemporary Indian chocolate production, he explains that they want to “keep authenticity [and] give back to that,” (pers. comm., October 31, 2023) hinting at the perceived exclusivity of koji originating from Japan. As such, they implement novel methods for growing koji in the micro-climates of their partner farms, even developing their own strains.

The value-added proposition that koji offers is twofold: first, koji offers the perception of quality by way of globalized commodities like miso and shoyu that evoke Japanese craft, and, second, it offers the novelty of koji as a processing technique parallel to how third-wave coffee may distinguish between washed, wet-hulled, or honey processes. Since this koji-fermented chocolate was developed in India, a country with presumably a shallow history of incorporating methods and terminologies from Japan, it seems that koji helps them align their product with the growing interest for artisanal and high-quality food products in India. Taken further, it reifies existing configurations of how a Japanese version of craft is imagined in these spheres. Localizing koji usage to the Indian context and “giving back to that” practice in Japan pays homage to it. One way this prestige projects onto the product is in their pricing, with the koji-fermented chocolate priced at more than three times anything else in their collection of single-origin chocolates.

**Figure 2:**
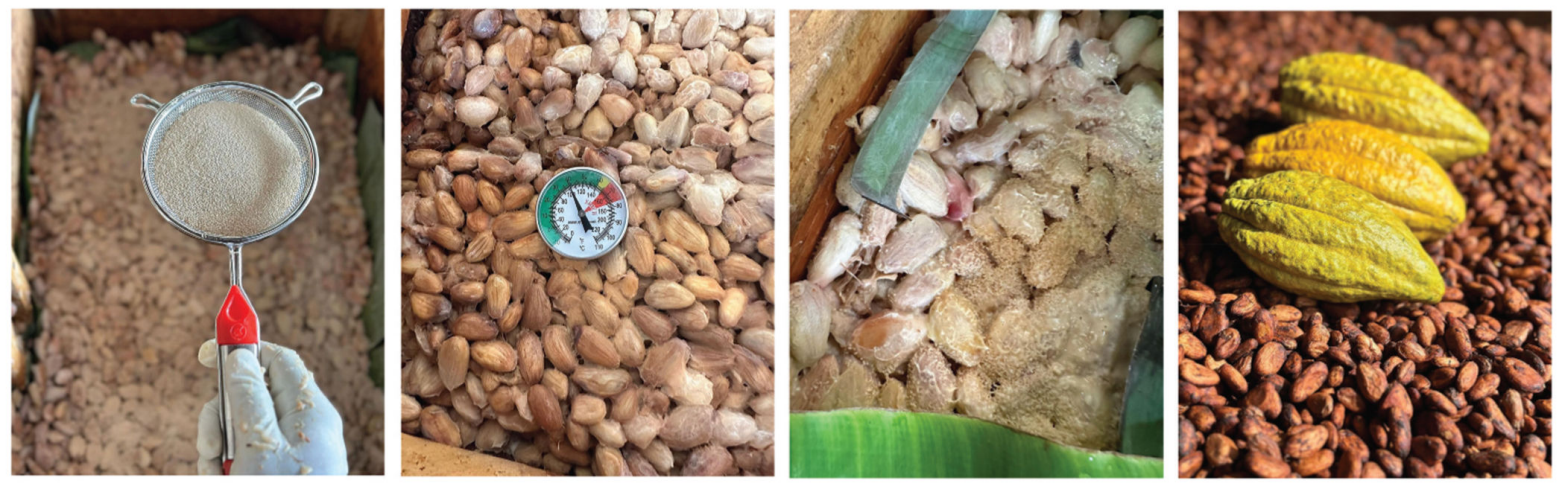
Koji fermentation of cacao. (photo credits: Subko, used with permission).

Koji would be an unfamiliar term for their consumer base, so their packaging and website take precautions to explain it. Their website describes koji as “a mold,” but also “a magical microbe” responsible for fermenting the cacao to produce the flavor profile in the final product (Subko n.d.). Notably, the koji-fermented chocolate falls under their Fermentation Fantasies series, a product line that relies on other fermentation processes such as a maceration technique and a double fermentation setup using passionfruit. Whereas these processes are driven by yeasts and lactic acid bacteria, the koji-based fermentation relies on introducing the fungus directly to the cacao pulp (Figure 2) and with it come material challenges (e.g., moisture and humidity balance) that the other processes may not need to consider, due to its fungal nature.

The package features elaborate instructions for how the cacao was fermented (Figure 1c). Against a lavender background, gold-colored writing in Hindu, Urdu, and Japanese repeat the product’s distinguishing feature: koji. Elaborate tasting notes and production steps of the fermentation process are enumerated. Detailed technical explanations about the fermentation process takes up most of the back side, with emphasis on the three layers of what they call “koji yeast,” cacao beans, and banana leaf. The label emphasizes the fermentation process over other steps in the chocolate-making process (e.g., conching or tempering steps). Thus, showcasing koji’s usage to this level of technical detail points to the possibility of koji functioning as shorthand for the producer’s care for craftsmanship while koji itself operates as an ambassador of high(er) quality. It may serve a similar function to the way Greg de St-Maurice refers to Kyoto’s heirloom variety of Shishigatani kabocha squash as “ambassador vegetables” which function as “a sign to consumers that [producers] carry high-end, distinctive, high-quality produce (2017, 295). For Subko to display this information above others, we argue, performs a kind of intense commitment to experimentation and branding.

The source of Subko’s koji knowledge stems from Gudi’s network in India. He developed an alternative koji-growing process – without Japanese koji rooms for temperature and humidity control – with the help of a professor in food engineering at an agricultural university nearby. While he did not fully disclose the strains used or the other ingredients included in the koji “yeast mix” listed on the packaging (Figure 1b), he notes a tension between Japan/Indian microbial contexts: “We want to develop our own [strains], we don’t want to incorporate the foreign strains into the Indian market[…]. That’s my concern, I don’t want to spoil this environment” (pers. comm., October 31, 2023). Subko’s use of koji (of Japan) to leverage the formation of a localized high-quality product (in India) creates a tension between foreign/threat and native/thriving microorganisms.

## Case study 2. Koji Mayo by Hakko Lab

Koji Mayo is a vegan alternative to egg-based mayonnaise launched in 2023 as a collaboration between Ine to Agave Brewery and Hakko Hub in Japan. Ine to Agave Brewery makes sake in rural Akita, which only recently was able to procure a new alcohol license for export. Hakko Hub is a multimodal online platform popularizing Japanese fermented foods through articles, in-person workshops, tours, and an online shop. Hakko Hub was founded by Takashi Sato, an eighth-generation tamari producer and president of the US-based family-owned brand San-J International. The Koji Mayo product is described as a “strategic co-brand launched with several food manufacturers” (Kimura 2023, para. 8) for the triple effect of: eliminating food waste from sake production in Japan, creating a vegan condiment destined for the US, and reviving the local economy of rural Akita.

Shuhei Okazaumi, the founder of Ine to Agave Brewery, first created the product using sake lees known as *kasu* (waste) in Japanese. Lees is a byproduct of the sake-making process, referring to the solids after the *moromi* (or proto-sake) liquid gets pressed. The lees still contain active enzymes because the moromi contains high amounts of koji-inoculated rice and because the by-product does not undergo pasteurization like its liquid counterpart (i.e., sake). Part of the impetus for using lees stems from the brewery concept of “turning foods with high disposal risk into treasures” and minimizing food waste (Ine to Agave n.d.-a). Lees is the main ingredient, so Koji Mayo does not contain egg like conventional mayonnaise (Ine to Agave n.d.-b). Notably, the alternative mayonnaise does not contain additional koji and only relies on the extant koji that was once part of the sake’s moromi.

The naming for Koji Mayo is perplexing on two fronts. For one, the product carries different names in different countries. It is currently only produced and available within the Akita prefecture through a production facility that sells the product as Hakko Mayo, with *hakko* being the general word for fermentation in Japanese. The product’s imminent expansion seems predicated on Hakko Lab’s consortium of international food manufacturers, including the founder, Takashi Sato.^2^ Eventually destined for the American market, the product curiously undergoes a name replacement of “hakko” for “koji.” Sato explains:

The product name in Japan is Hakko Mayo. However, when I solicited opinions from many people in the U.S., many of them said that the word “Hakko” is too minor and should be avoided. Therefore, I decided that Koji Mayo would be more suitable for future sales in the US. Kasu Mayo was also a candidate, but the word “Kasu” was not well known, and while using the by-product Kasu as the product name was sufficient to convey the fact that it was an upcycled product, there were a certain number of people who did not like the idea of using a raw material that is normally thrown away. (pers. comm., November 13, 2023)

**Figure 3:**
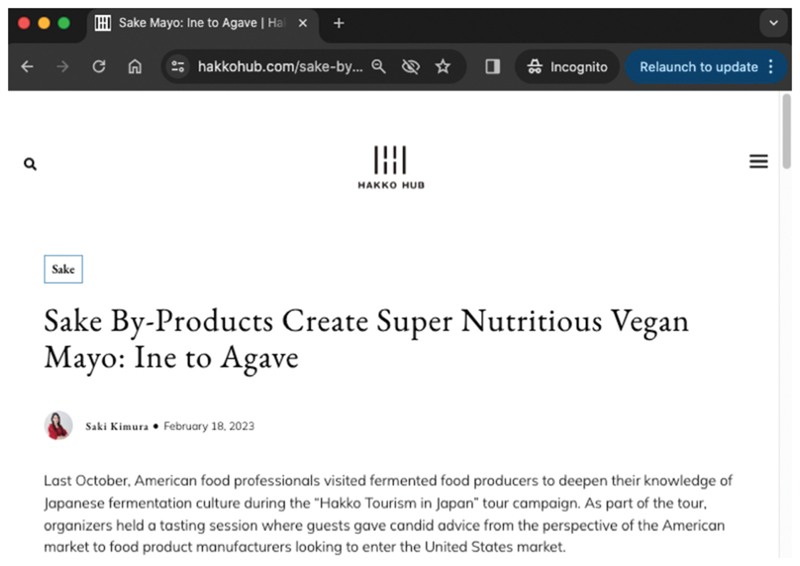
Hakko hub website.

**Figure 4:**
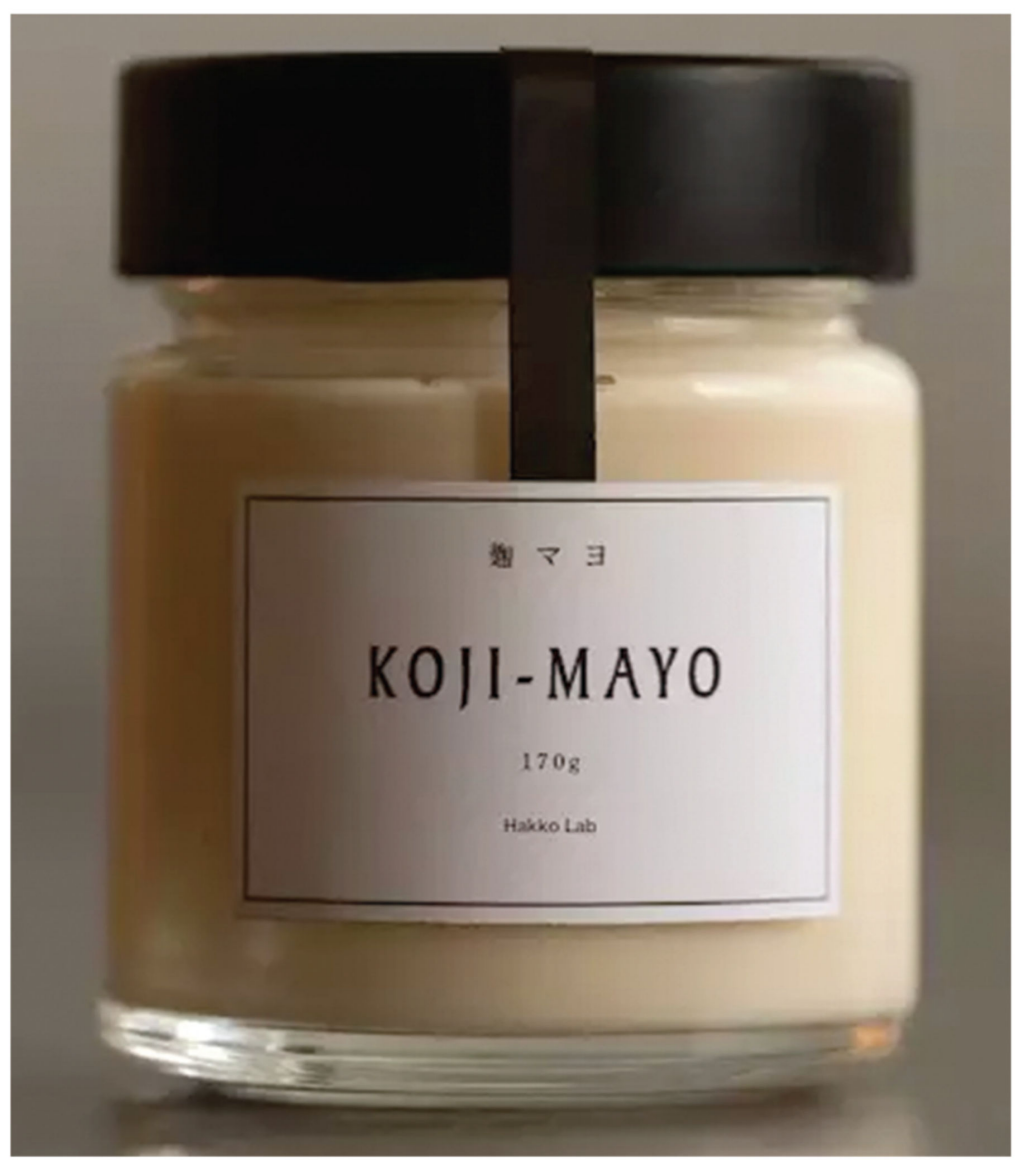
Packaging of Koji Mayo.

In the meantime, the product may have had an intermediary name of Sake Mayo as seen on the page name of their website (Figure 3); however, after considering the sales potential of this product in the US. The latest name of the product distributed via Hakko Hub is Koji Mayo (Figure 4), with a minimalistic white label featuring the name in both English and Japanese.

Sato’s description demonstrates how the term “koji” functions as a useful marketing tool. In this instance, the term “koji” is affixed to mayo, which frames the product as a type of mayonnaise; yet the product is explicitly named as a kind of koji-based condiment, when other such condiments like miso or shoyu do not include koji in their naming. (For comparison’s sake, even the tamari sold by San-J neither uses koji in their naming nor lists koji in their ingredient list even though it is a key component.) In a Western context, the term’s allure seems to parallel the popularization of koji throughout a North American and European culinary scene. In fact, Sato credits the global success of koji to haute cuisine: “Thanks to the achievements of Noma in Denmark, there is a growing interest in koji fermentation among top chefs in the United States and Europe” (pers. comm., November 13, 2023). He acknowledges that this familiarity inheres to a niche consumer base, admitting that the product may be “specialized for gourmets [グルメ、or gourmands]” (pers. comm., November 13, 2023), which may explain the targeting of a (US-based) market flush with mayonnaise options and a (vegan) consumer base invested in ingredients and taste.

According to Sato, “Japan was the only country that has actively managed and used koji fungi for fermentation, so in this sense, it is a material unique to Japan” (pers. comm., November 13, 2023). We find evidence on the contrary, discussed in the following section. But it is in this context that Sato sees koji as a processing technique that opens up a world of possibility for Sato and his collaborations:

The fermentation technique using koji is *just a method* and can be applied to a wide variety of materials. Many Japanese brewers are conservative and use only traditional ingredients, but we take the stance that we must be flexible and change when different countries demand different qualities[…]. Just last week, we collaborated with Eleven Madison Park [a U.S. restaurant holding three Michelin stars] to develop Corn Shoyu and Black Bean Tamari. (pers. comm., November 13, 2023, emphasis added)

By thinking about and using koji as a technique to deploy, flexibility brings in new substrates like corn, black beans, and green peas to produce new ferments. Sato himself witnesses such flexibility with his company San-J International, even emphasizing how adaptation to the American context was crucial to “incorporate our Tamari into the American dinner table more easily. It didn’t matter to us if it wasn’t used in the same way as in Japan—we had to evolve to bring our product to a new market” (San-J. n.d.).

Viewed as a tool, koji creates opportunities for upcycling food waste (like sake lees), which Sato sees in the greater context of social responsibility. That said, it remains questionable whether the scale of upcycling with koji can be effective. For instance, within the Hakko Hub oeuvre, upcycling with koji at a household level is considered “something anyone can do in their own home” (Shockey 2023, para. 17), adding that the practice “is a cost-effective, natural, safe, delicious, and nourishing way to give life to food waste” (para. 9) – yet, the article assumes one has unencumbered and consistent access to koji. Using a similar logic, Sato recounts a call over social media for making pumpkin *amazake*, made from combining koji with the large amounts of pumpkin discarded after Halloween. With an eye toward developing “other products and contribute to society” Sato notes how his involvement with San-J and Hakko Hub synergizes around this form of social impact (pers. comm., November 13, 2023). It is as if the metonymy of koji as Japan allows these producers to invoke a Japanese technique that gives back to the world.

## Case study 3. Fermented hot sauce by sheldrake & sheldrake

In 2022, Merlin Sheldrake and his brother Cosmo Sheldrake launched a koji-based hot sauce in the UK. Rooted in biology, Merlin studies fungi as an ecological, philosophical, and relational entity that highlights how we live in interdependent, indeed entangled, worlds (Sheldrake 2021). Cosmo identifies as “an ecological sound artist” (Sheldrake & Sheldrake n.d.). Their hot sauce was the result of an ongoing enthusiasm they cultivated after being exposed to a family friend’s fermentary in the US. Visits there inspired several household attempts at making hot sauces as Christmas presents. During the COVID-19 lockdown, they scaled up to a business but recount how “it was less a kind of ‘let’s try and make our living through hot sauce’ but more about having a shared art project together, that could be fun” (Merlin Sheldrake, pers. comm., January 16, 2024).

Their product also celebrates a network of relationships that bring together kin, friends, and fungal catalysts. Their main supplier for koji, Jonathan Hope of Kultured London, began collaborating with the Sheldrakes after a chance meeting at a book launch where the three of them met through their fathers. Hope explains that the recipe took almost two years to become what it is today, from the time the brothers approached him (pers. comm. November 22, 2023). As a group, they seemed enamored by the idea of augmenting an existing hot sauce recipe with the koji fungus, given Merlin’s research area and recent book.

**Figure 5:**
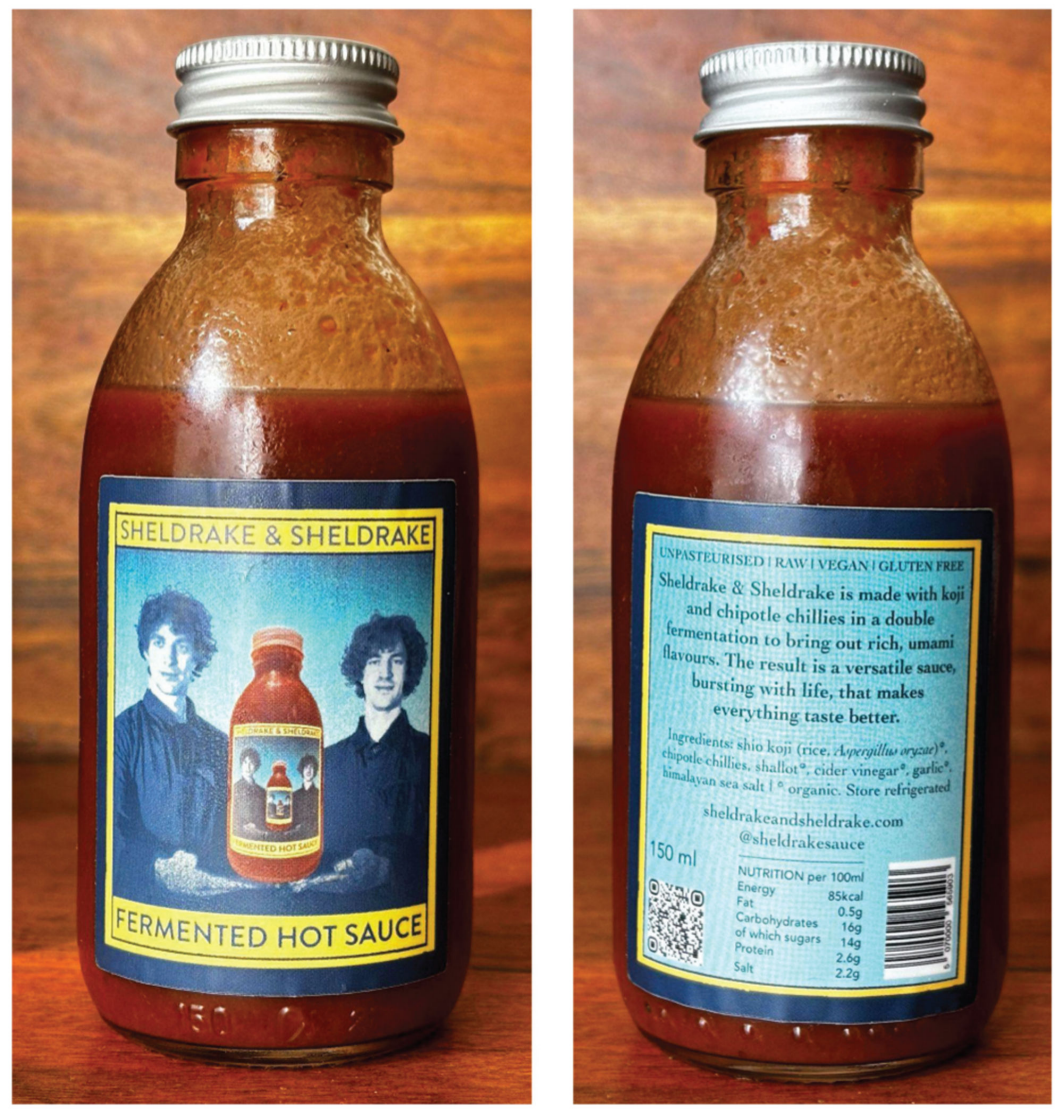
Packaging of Sheldrake & Sheldrake fermented hot sauce.

Unlike the other two case studies, koji is not specified upfront in the product name or series. Rather, koji is listed as an ingredient in the form of *shio-koji* (Figure 5b). Shio-koji is a salt-based condiment that takes ready-to-use koji and ferments it further with salt and water, which is sometimes used in Japanese cooking (e.g., meat/fish seasoning). In this sense, the Sheldrake’s hot sauce combines two ferments into one, mixing the lactoferment of chili peppers and the shio-koji into what they call a “double fermentation” (Merlin Sheldrake, pers. comm., January 16, 2024). To make koji legible to their consumer base, an explanation of their recipe provides accessible and relatable examples:

The saucery takes place in a two-stage process. First, we make koji by introducing a specialised fungus to rice (koji is an ancient fermented food that forms the basis of miso and soy sauce). We then add the koji to a brine containing chillies, onions, and garlic, where it undergoes a lacto-fermentation. (the process that creates ferments like sauerkraut and kimchee) (Sheldrake & Sheldrake. n.d)

To introduce their audience to koji, they use taste associations by name-dropping fermented foods more familiar to a Western audience, such as miso, soy sauce, sauerkraut, and kimchi.

The label features a creative photo print of the brothers holding their hot sauce, which aids in making koji appear approachable to Western markets (Figure 5a). On the bottle’s reverse, a label explicitly names shio-koji as the first ingredient (Figure 5b), specified further to comprise organic rice and *A. oryzae*. The label also emphasizes deliciousness, stating that “the hot sauce these microbes create makes everything taste better” (Sheldrake & Sheldrake, n.d.). By connecting the “specialised fungus” to enhanced flavor, koji becomes the microbial shorthand for delectable storytelling.

Their emphasis on koji’s flavor potential over its fungal nature seems deliberate. The Sheldrakes justify using the term “koji” due to the aversion that people can have toward the idea of molds and fungal spores transforming their foods: “We tried to say koji or a cultured rice with *Aspergillus* mold but [people] might not be as comfortable with some indigenous mold and it’s not going to be such a turn on” (Merlin Sheldrake, pers. comm., January 16, 2024). Instead, they leverage what they call the “gourmet gastronomic experimentation happening around the world” to highlight the taste potential of this particular mold (Merlin Sheldrake, pers. comm., January 16, 2024). They attribute koji to being the distinguishing ingredient in their product:

The same recipe without koji has a very, very different flavor profile, so we wanted to talk about what goes into building the flavors […]. You have lacto- and yeast ferments, and basically in my mind, this is like the next level up. The possibilities of explorations you can end up with, the way you can transform things… I was speaking to a friend who worked at Noma and it seems that koji unlocks *a whole new world of possibilities*. (Cosmo Sheldrake, pers. comm., January 16, 2024, emphasis added)

This whole new world of possibilities aligns with the rise in koji’s popularity across the world driven by gastronomic interests. Consistent with what Heather Paxson and Stefan Helmreich call a “microbial moment” (2014, 166), Cosmo notes how ferments serve “as one of those cultural moments [with] this real sense of the connection across time and the continuation, whether it’s microbes, yeast or whatever” (pers. comm., January 16, 2024). The Sheldrakes seem to view the cultural moment of microbes as something that offers continuity from historical praxis to contemporary food innovation.

Alongside their niche interests in fungal ferments, their product acknowledges both the historicity of Japanese koji and the recency of koji momentum in the culinary sector: “We wouldn’t know about koji unless it had been celebrated and used in Japan for so long; a celebration not just of Japan but celebration by a diasporic world of gastronomes, like Noma is a big example” (pers. comm., January 16, 2024). At a time when gastronomes are accused of cultural (and culinary) appropriation, one of the brothers explicitly states that as the spores are “sourced directly from Japan […], it is directly plugged into a deeply rooted cultural practice and value that *we’re borrowing from*” (pers. comm., January 16, 2024, emphasis added).

## Analysis and themes: strategic storytelling, gourmandization of koji, and human-microbe labor relations

The three case studies demonstrate that koji usage reflects both material changes (i.e., as a processing technique) and discursive ones (e.g., as a branding strategy). Across our three cases, koji ranges from a fungal spore in an experimental process (Subko chocolate), to a tool for upcycling food waste (Koji Mayo), to an ingredient for taste-making (Sheldrake hot sauce). For all three, koji symbolizes gastronomic value in a cultural moment that embraces microbial possibility, where companies selectively mobilize the term to convey global status, social responsibility, or deliciousness.

Our analysis points to three themes that feed into each other: strategic storytelling, gourmandization for product distinction, and human-microbe labor relations for a purportedly better world.

### Theme A: strategic storytelling

Koji enables strategic storytelling for all three cases: it enables a chocolate to become novel and legible as a high-quality product, it provides opportunities for a mayonnaise alternative to revive a rural economy and expand vegan repertoire, and it characterizes a paradigm shift where molds can transform mundane ingredients into tasty hot sauces. In comparing when and how these products name koji relative to its material use, we see that timing matters in such stories. Case 1 consistently refers to koji from inputs to throughputs to outputs, even if it presents erroneous information (e.g., calling koji a yeast instead of a mold).^3^ Their insistence shows how koji is the key feature of the product, as if to claim the niche market (at least in India) and lead the way with third wave cacao (or whatever would come after bean-to-bar). In Case 2, Hakko Lab use the name “koji” instead of lees/kasu, the latter of which is the actual ingredient for their mayonnaise substitute. Their mention of koji is only on the final product label, chosen via crowdsourcing, based on koji’s recognizability (at least for the culinary sector) and its ability to connote an alignment with zero-waste movements. In contrast, the hot sauce in Case 3 does not center koji in its name, and only names koji in the form of the ingredient shio-koji, without any other mention of it beyond its taste-making abilities. In fact, koji is defined by its proximity to other ferments mentioned on the label, seemingly in an attempt to create a more taste-forward appeal for the fungus and make the condiment more enticing to a wider consumer-base. In each of the three cases, when to name koji is just as important as the terminology used in their respective storytelling tactics.

Our case studies span different geographic, cultural, and economic contexts, but the term “koji” curiously still applies across all. Linguistically, people could have called koji a “Japanese malt,” since it is a historic term that still receives occasional use (e.g., by the KIOKE group in France and WaNaHong in the UK). The malt reference to koji harkens back to the introduction of foreign alcohols toward the late 19^th^ century, when German beers and Scottish malt whiskeys were entering the Japanese foodscape (Francks 2009). In a brewing context, koji functions similarly to barley malt as it breaks down the starches into fermentable grains, but as British chemist Robert Atkinson remarks in a footnote, “I know of no single word by which *koji* might be rendered into English” (Atkinson 1878, 315). Koji has no equivalent in English, argues Atkinson, who distinguishes koji from malt based on how they derive their enzymes (malt develops amylases endogenously by mobilizing what already inheres to the grain, whereas koji relies on an external substance – a fungus – that grows and converts the starches into amylase). So while *the thing itself* enables the same use-cases, the material reality (i.e., enzyme source) differs enough that, at least for Atkinson, the two terms ought to remain distinct. That our case studies use “koji” and not “malt” suggests a tactic for placing their products in a story of Japanese-inspired food futures intended to capture the attention of globally minded consumers of today.

Even at the species level, koji has a history of strategic storytelling consistent with the positive spin seen in our three cases. The year 1960’s aflatoxin scare of *Aspergillus flavus* (a relative of koji’s most common fungus *Aspergillus oryzae*) raised questions regarding koji’s safety in the food supply as both fungal species share the same genus and functional group. The scare resulted in deliberately separating the two species, both in terms of microbiological category and narratives of control. Lee (2019) describes how scientists crafted an evolutionary narrative whereby *A. oryzae* was cast as a nontoxic, domesticated species that could *only* come from Japanese breweries, which equated domestication with safety. This contrasted with *A. flavus*, depicted as a wild, toxic strain naturally found in rice fields. Furthermore, Lee (411) notes a double standard for how news outlets spread the fear about aflatoxins being found in the home kitchen (e.g., homemade miso using wild koji from paddies), yet no issues were raised against the Japanese fermentation industries. Over decades, Japanese efforts to reclassify koji *as specifically A. oryzae* excluded aflatoxin-producing *A. flavus* in a discursive sense, which then strengthened the spirit of experimentation with *A. oryzae* today. Especially between the cacao trials in Case 1 and the two-year recipe iteration in Case 3, we see koji framed as the safe and worthwhile mold with which one can experiment, especially in the name of taste.

Add to this another layer of koji as a Japanese mold, and we see an elision of koji as signifier of Japanese taste with gourmet taste.

### Theme B: gourmandization of Koji

Experimentation feeds the gourmandization of koji, especially since iterating for elevated tastes can conflate culinary value with place-based distinctions. Across Cases 2 and 3 Michelin-star restaurants (e.g., Noma, Eleven Madison Park) are mentioned as inspiration or justification to mobilize koji. This gourmandization conflates the cultural clout of Japan as it is perceived by koji-makers from abroad (Evans and Lorimer 2021) with the culinary prowess of Japan in the “global hierarchy of dominant culinary discourse” (Ray 2016, 122, see also Wank and Farrer 2015). Whether phrased in terms of “giving back to” Japan (Case 1) or “borrowing from” Japan (Case 3), it seems that Japanese cuisine encodes the aspirations of “food adventurers” (Heldke 2003, xiv) who depart from the default and cater to those who “eat their way around the world.”

All three case studies use taste to forge cultural-economic relations involving multiple countries (i.e., India, UK, Japan, and US), confirming Giddens (2013) notion that globalization creates new contact zones within and across nations. Whether at the level of branding strategy (Case 1), consumer targeting (Case 2), or spore sourcing (Case 3), koji inherently draws transnational intrigue that help construct koji’s gourmet profile. In Case 1, Gudi incorporates koji-practices amidst the native microbes found in Indian terroir, “keeping authenticity” while “giving back” to the Japanese culture, as if the Japanese were the sole cultural owners of the fungus and its know-how. The presumed cultural ownership of Japan over koji is reinforced by comments in Case 3, where the producers consider themselves to be “borrowing from” bits of Japanese heritage. In both instances, authenticity seems to be taken at face value, used as a signpost for claiming (Japanese) craftsmanship but without attending to its (intra-Asian or Eastern/Western) relations brokered by the likes of race, gender, and class.^4^

Sato of Case 2 makes explicit that koji is a “material unique to Japan” although we find evidence to the contrary. Choi (2023, 712) notes how staple ferments in Korea like soy sauce and *doenjang* rely on fungal starters and make up the key flavors of temple food. Shih and Umansky (2020, 40) outline the varied ways of using koji in fermentation contexts across East Asian food cultures, including *qu* in China (which ferments to become *ouche* and *doubanjiang*) and *meju* and *nuruk* in Korea (which ferments to become *gochujang, makgeolli*, and *soju*). Against this evidence on the complexity of koji’s origins and applications, Sato’s claim that koji is unique to Japan suggests a construction of Japanese identity *in* koji (i.e., as seen in culinary heritage) and subsequently *through* koji (i.e., through the use of koji in global settings). Koji-the-ingredient is seen as being exclusive to Japan, while koji-the-enzymatic-toolkit can be exported and utilized elsewhere. So while Koji Mayo uses lees (a by-product in sake brewing) that happens to contain some traces of koji, centering the name “koji” seems to leverage glocal pathways that evoke and connote haute cuisine and culinary prestige. In turn, koji becomes a strategic device to gain a foothold in the global marketplace, becoming, like Neopolitan pizza, a “product which is rooted in a local and traditional know-how, and which can be traded, at the same time, on the different markets of culture, tourism, catering and communication” (Stazio 2021, 415). By encoding koji as being markedly Japanese, these case studies can convey notions of exceptional taste.

Gourmet terms help equate koji with exceptional taste, which, in turn, helps frame it as an exceptional fungus, shifting its schematic category from a cautionary mold to something esoteric and specialized. Especially for Cases 1 and 3 which are based outside of Japan, the products overwrite koji’s fungal aspects by using embellishments like “magical microbe” to describe chocolate-making or spotlight the “double fermentation” that “makes everything taste better” when making the hot sauce. Western paradigms’ associations of fungi and mold with spoilage and rot help explain the products’ attempts to replace these connotations with descriptors that are novel, innovative, and delicious (see also Dunn and Sanchez 2021). It seems that fungal ferments produce what material scientist Johnny Drain (2021) calls an “axis of disgust and deliciousness” because of their simultaneous semiosis. Claude Fischler’s concept of neophobia and neophilia may also explain the repositioning of fungal ferments (Fischler 1988, 954) as it pushes the boundary of Paul Rozin’s notion of omnivorousness (Rozin 1997), where consumers can choose between food as forms of identity confirmation (e.g., third-wave, vegan, and zero waste), for which they are willing to pay extra. Notably, all three products were priced at more than triple the average price of that commodity, making koji a value-added process and ingredient. These ferments are, after all, fermented by choice, not necessity.

These products have been sold out and re-stocked, indicating a clear market of consumers who are willing to pay for the perceived gourmet characteristics. Backed by its Japanese association, notions of terroir, or the zeitgeist in the current microbial moment, koji-based products invite consumers to embody a sense of distinction that steers them away from the commonplace. Consistent with previous food studies literature on taste-based distinction (Johnston and Baumann 2010, Naccarato and LeBesco 2012), koji practitioners negotiate the sellability of their products by selectively mobilizing koji to signal high quality. Driven by consumer choice and competitive commoditization, it appears that the gourmandization of koji both feeds and benefits from capitalist notions of individualism, (microbial) labor exploitation, and (human) exceptionalism.

### Theme C: human-microbial labor relations

Experimenting with koji to create novel products presumes a level of microbial expendability common in how human-microbe relations are imagined under capitalism. Especially since modern industrialization, microbes tend to fall into categories of utility (e.g., helpful, harmful) which place humans in “stewardship of” microorganisms (Felder, Burns, and Chang 2012, 64). In food settings, microbes are often sorted by their promised ability to carry out enzymatic work; however, microbes are neither machines nor predictable, so a mechanistic understanding of them often brings about more tensions than seamless means of production (for agricultural examples, see Turner and Szymanski 2023). Such mechanistic assumptions set up a paradigm of human control that flattens microbial agency into something that one *works on* versus living with microbes (Hey 2022), the former of which supports greater projects of commodification. Indeed, Rath (2023, 40) recognizes koji as “a fungus with its own agenda that may not always align with the people trying to mobilize it.”

It is worth noting that all of our case studies rely on small-scale use of koji, whether in the form of a lead experimenter (Gudi in Case 1), a single brewery (Ine to Agave in Case 2), or an outsourced collaborator (Jonathan Hope in Case 3). Here, individual practitioners make up the knowledge-base for koji production, including how to set up, manage, and work through the hyper-curated micro-climates necessary for a fungal ferment to develop over the course of 3–4 days. In other words, making koji is not a “set and forget” kind of ferment; materially speaking, the success of koji production hinges on meticulous tinkering of temperature and humidity over time, such that the inner starches of a substrate (e.g., cacao, rice) transform into key enzymes for later use.

But even at small-scale production, microbes are leveraged to produce capital for (selective) human gain. For at least these practitioners, human labor entails fostering environments for Aspergillus to thrive, while microbial labor entails digesting substrates into enzymes. (And, later still, more human labor is brought in to commoditize and distribute the koji product.) In an analysis of soil microbes and agricultural production, Krzywoszynska (2020) argues for seeing these labors and resources as being co-constituted in more-than-human ways: while microbes make better soils, humans make conditions for better microbes in said soil. For instance, microbes make soil nutrients (like cobalt) available to plants, which become feed for animals who become livestock and therefore assets. In a fermentation setting, microbial labor makes better resources for humans (as food to sell, to eat, to live), who in turn make better conditions (i.e., make food-resources better available) for microbial transformation. The materiality of these co-constitutive labor relations is crucial, in that it “means that […] such manipulation occurs at the level of ecosystems rather than at the level of individual bodies” (239). While Krzywoszynska here refers to individual cells of microbiota, we extend the argument to include individual human bodies as well, such that koji comes into being as a result of multiple human-to-human relations (such as the brothers’ network in Case 3, the international business configuration in Case 2) in addition to human-microbe networks that make koji-making practices possible. That said, given how material processes for working with fungal ferments (like koji) are especially finicky and thus require specialized know-how, we can presume that koji-based tacit knowledge *came from someone* who was capitalized upon, somewhere in the (hopefully equitable) transfer of fermentation practice. Here, we call attention to the tension between the exclusivity of koji spores, products, or know-how and the growing aspiration to spread koji-use globally.

However it is that we choose to parse these co-constitutive labor relations, human-microbe enmeshments show *who* or *what* is at stake. Heldke (2018) calls out “symbiosis” as a rose-colored myth and instead argues for thinking about human-microbe interdependencies as relations with teeth. Microbes can cause foodborne illness to incapacitate a person but gut microbes are also responsible for turning food we ingest into something we can absorb – there is no conclusive or evermore settled relation other than we are, and have been, constantly using each other to continue making a life/living. Similarly, in writing about methane emissions generated from cows and their rumen, Folkers and Opitz (2022) show that more-than-human thinking in capitalist ventures requires an understanding of “interlaced and convoluted sets of living things” (345) whose inter-twined metabolisms reveal a politics that “is never innocent. After all, it is about metabolic relations that involve digestion and colonization, eating and being eaten, mutualism and parasitism” (345). In other words, human-microbe relations in and through koji always cast shadows of power and exploitation that can – always – stand to be examined and reexamined.

The three themes intersect with each other such that the material practices of working with koji-spores can determine the amount and quality of koji-enzymes and what the koji-ingredient *does* to or for a food product, which further impacts how it can be narrativized and/or strategically deployed in gourmand discourses and global marketplaces.

## Conclusions and future areas for research

In this paper, we have analyzed three case studies that use koji outside of conventional contexts, with specific attention to how and when koji is used materially and discursively. We analyzed a koji-fermented chocolate, a lees-based mayonnaise-like condiment that claims koji in its name, and a quirky hot sauce that combines shio-koji with lacto-ferments for a double punch. In all three cases, koji comes to matter (A) in selective storytelling to strategically foreground or background its usage, (B) when evoking existing forms of gourmet-inflected social distinction, and (C) as a material resource to expend for culinary exploration, economic expansion, and global capital gain.

Indeed the excitement for koji’s transformative abilities lends itself to increasing democratization, but its hype *coupled with koji’s ambiguity* as sometimes-spore, sometimes tool, sometimes ingredient, obscures the knowledge politics that inhere to koji-making. As such, we encourage future research that traces the lineages of koji knowledge with attention to the raced, classed and gendered labor of knowledge transfer. Attention to what stabilizes these knowledge-based networks will be crucial to understanding who stands to gain and who can claim to know koji practices. We hope for future studies that parse how, exactly, race, ethnicity, age/generation, able-bodiedness, colonial histories, and language ability can color the quality of knowledge or render it extractable by a select few. Given the rampant history of “food adventurers” parachuting into East Asia and extracting cookery know-how, we are particularly wary of how terms like “authentic,” and “novel” stand in for “othering” (Heldke 2003). If indeed, as Evans and Lorimer diagnose, “kōji is taking over the world,” (S372), then we follow their claim with: who gets to be a part of that story when people (usually men, usually chefs, usually cosmopolitan) continue to invisibilize their sources?

The ambiguity of koji as a term, in turn, informs its practices, such as hyper-experimentation, glocal commerce, and performative labeling of value-added foodstuffs. So as koji continues to manifest across the world, with practices and terminologies outside of its cultural context, we would do well to stay attentive to its potential for fetishization. In particular, the hype about experimenting with koji may appear (or even *be*) promising, especially in terms of its potential to transform flavor and reduce waste. But aiming for possibilities can also occlude the extant knowledges for how to work with fungal life and overshadow attempts at keeping such knowledge continuous, multi-plicitous, and whole from one generation to the next. This is not a critique against adaptations; it is a question of whose practices persist and are seen as valuable.

Today’s fermentation is often practiced out of choice, which is tied up with capitalist notions of accumulating resources – both tangible and intangible – to serve the ever-changing expectations of the contemporary consumer. Crucially, this resource accumulation benefits from ambiguity, in that *Aspergillus* and its know-how remain in the hands of the monopolized manufacturing in Japan, while the foodmakers who can afford to learn and grow koji appear to have the financial capacity to travel there, procure ingredients and tools, or gain knowledge from individuals with firsthand experience. If it is a classic story of (culinary) haves and have-nots that we are after, then we have already arrived. For other worlds to come into existence, we, the fermenters, will need to reckon with how we dole out, oversee, witness, partake, honor, cite, and continue to broaden knowledge sharing.

Lastly, continuing to view any microbe as a tool continues to drive an onto-ethico-political myth of human exceptionalism. This mentality also exacerbates the ambiguity because human control flattens the microbial agency into a kind of sameness where koji-the-spore operates in the same way as koji-the-ingredient, when they carry differential stakes to both human and environment. These hierarchical distinctions are arbitrary and serve to justify human exploitation of other beings, including microbial life, for the benefit of select humans who can afford to make, purchase, and eat fungally fermented innovations. By challenging the boundaries constructed by humans’ attempted superiority over microbes as passive agents or mere tools, we acknowledge the significance of their agency in the co-creation of fermentation and the sustainability of our multispecies world.

## Notes

1 Subko chose a company name that is a homonym to *sabko* in Hindi, a word meaning “for/to everyone.” In this light, their efforts to connect Japan and India also emphasize an aspiration to produce chocolate at the level of a pan-Indian subcontinent.

2 As of this writing, Hakko Lab does not yet have its own public-facing identity beyond that which was facilitated through Hakko Hub. Based on the scope of business, it does not seem affiliated or to be an extension of the Hakko Labo in Yamanashi led by Hiraku Ogura.

3 Koji is not a yeast; it is a type of filamentous fungi which grows by extending out a filament, which can be seen in the form of hyphae/branches when the koji clumps together. In comparison, yeasts are a single-celled fungus that grow by budding off one cell into two.

4 While our study and research instrument were not designed for it, the topic of gastrodiplomacy may offer insights on who benefits from these power relations.
